# Thermosensitive TRP Channels Are Functionally Expressed and Influence the Lipogenesis in Human Meibomian Gland Cells

**DOI:** 10.3390/ijms25074043

**Published:** 2024-04-05

**Authors:** Melina Keller, Stefan Mergler, Aruna Li, Ingrid Zahn, Friedrich Paulsen, Fabian Garreis

**Affiliations:** 1 Department of Functional and Clinical Anatomy, Friedrich-Alexander-Universität Erlangen-Nürnberg (FAU), Universitätsstraße 19, 91054 Erlangen, Germanyfriedrich.paulsen@fau.de (F.P.); 2 Department of Ophthalmology, Charité—Universitätsmedizin Berlin, Corporate Member of Freie Universität Berlin and Humboldt-Universität zu Berlin, 13353 Berlin, Germany; stefan.mergler@charite.de (S.M.); aruna.li@charite.de (A.L.)

**Keywords:** dry eye disease, meibomian gland, meibomian gland dysfunction, TRP channels, thermo-TRPs, tear film

## Abstract

While the involvement of thermosensitive transient receptor potential channels (TRPs) in dry eye disease (DED) has been known for years, their expression in the meibomian gland (MG) has never been investigated. This study aims to show their expression and involvement in the lipogenesis of the MG, providing a possible new drug target in the treatment of DED. Our RT-PCR, Western blot and immunofluorescence analysis showed the expression of *TRPV1*, *TRPV3*, *TRPV4* and *TRPM8* in the MG at the gene and the protein level. RT-PCR also showed gene expression of *TRPV2* but not *TRPA1*. Calcium imaging and planar patch-clamping performed on an immortalized human meibomian gland epithelial cell line (hMGECs) demonstrated increasing whole-cell currents after the application of capsaicin (TRPV1) or icilin (TRPM8). Decreasing whole-cell currents could be registered after the application of AMG9810 (TRPV1) or AMTB (TRPM8). Oil red O staining on hMGECs showed an increase in lipid expression after TRPV1 activation and a decrease after TRPM8 activation. We conclude that thermo-TRPs are expressed at the gene and the protein level in MGs. Moreover, TRPV1 and TRPM8’s functional expression and their contribution to their lipid expression could be demonstrated. Therefore, TRPs are potential drug targets and their clinical relevance in the therapy of meibomian gland dysfunction requires further investigation.

## 1. Introduction

Hundreds of millions of people worldwide suffer from dry eye disease (DED), one of the most common reasons to consult an ophthalmologist. This ocular disorder shows prevalences as high as 75% amongst adults over 40 years of age, with women more frequently affected. DED is linked to eye discomfort, itching, tiredness, burning, photophobia, redness, pain and visual disturbances. A large number of patients additionally suffer from decreased vitality, depression and an overall restriction of their daily activities [1,2,3,4]. In 2011, the International Dry Eye Workshop Study Report (DEWS) defined DED as “a multifactorial disease of the ocular surface characterized by a loss of homeostasis of the tear film, and accompanied by ocular symptoms, in which tear film instability and hyperosmolarity, ocular surface inflammation and damage, and neurosensory abnormalities play etiological roles” [5]. DED can be divided into two main categories: aqueous deficient dry eye—ADDE; and evaporative dry eye—EDE, mainly caused by the dysfunction of either the lacrimal or meibomian glands (MGs) [6]. The leading cause of DED, in particular EDE, is a dysfunction of the meibomian glands (meibomian gland dysfunction—MGD), which provide the outer lipid component of the tear film, function as a barrier that prevents the tear fluid from evaporating and are part of the formation of a smooth optical surface [7]. The MGs are situated in the tarsal plate of the upper and lower eyelids as individually branched alveolar sebaceous glands [6]. Each MG consists of one central duct with approximately 10–15 acini, opening out into the excretory duct with the orifices located in the lid margin, excreting lipid-containing meibum. The ducts are lined with squamous keratinized ductal epithelial cells, while the acini are made up of meibocytes arranged in functional secretory units [8]. They excrete the meibum through the holocrine secretion mode, so every MG needs specialized stem cells to replace the meibocytes lost in this process [9]. The maturation process of the meibocytes can be closely followed and divided into four stages: basal, differentiating, mature and hypermature. They migrate from the periphery to the acinus center, increasing their lipid content along the way [10]. Changes in the quantity or quality of the meibum result in instability of the tear film leading to increased evaporation, and this can be followed by hyperosmolarity, inflammation and pain [11]. The pathology behind MGD is only partially understood. Histopathological changes, such as cystic dilation, atrophy and granulation, seem to be the result of obstruction [12]. Hormonal changes, especially of sex hormones, also show an influence on meibum production [13]. The gold standard for the treatment of DED or MGD are various types of artificial lacrimal fluids to replace the missing tear fluid which is a symptomatic treatment but does not solve the actual pathologies. In recent years, the treatment strategy has slowly shifted to modifying the underlying mechanisms such as through inflammatory mediators and neuronal regulation [14].

Transient receptor potential channels (TRPs) are prominent focuses of research across many different pharmacology fields currently, including DED [14,15,16,17,18,19]. They make up a super family of 29 ion channels that can be divided into six subfamilies based on their amino acid sequence homology. They have polymodal activation properties and can be activated by various stimuli such as pH, temperature, pressure, voltage, chemicals, proteins or lipids. Classically, they are considered only in the context of neurons and sensory physiology because of their involvement in gustatory and mechanosensation, and noci- and thermoconception [20]. In contrast, TRPs are also functionally expressed in non-excitable cells of the lacrimal apparatus including corneal and conjunctival epithelial cells [21,22], in which they can release pro-inflammatory cytokines [23]. They are also involved in other signaling pathways: from apoptosis [24] to cell proliferation [25], differentiation [26] and migration [27], and angiogenesis [15,28,29]. Not all TRPs can be activated by temperature, as there are only six capable of heat or cold sensation [30,31,32]. All six of these temperature-sensitive TRPs (thermo-TRPs) have been identified on the ocular surface: the heat receptors TRPV1, -2, -3 and -4, as well as the cold receptors TRPM8 and TRPA1 [21,33,34]. In nerve terminals and corneal cells, TRPV1 and TRPM8 show a direct influence on basal tear production [15,33]. Furthermore, they also function as nociceptors and play a key role in the pain sensation that accompanies DED. In previous studies, we showed the expression and function of thermo-TRPs in various cells of the lacrimal apparatus and the ocular surface [15,21,29,34,35,36,37]. Their activation could potentially exacerbate conditions such as corneal dystrophies, but this is also associated with proliferation and migration, which are central to wound-healing processes; for example, the up-regulation of TRPV1 in human pterygium epithelial cells modulates EGF and VEGF responses [38]. As a result of these properties, TRPs are considered highly interesting drug targets for the treatment of DED. It is important to find out whether these drugs could have side effects on other tissues of the lacrimal apparatus in addition to their effect on the ocular surface. The expression of thermo-TRPs in the human meibomian gland is still unknown. In the present study, we therefore investigate the expression and functionality of thermo-TRPs in human meibomian glands, and a possible connection with lipogenesis in meibocytes. These results could form the basis for further investigations of novel TRP pharmaceuticals for the treatment of MGD and DED.

## 2. Results

### 2.1. Temperature-Sensitive TRP Channel Expression in Meibomian Glands

**Figure 1:**
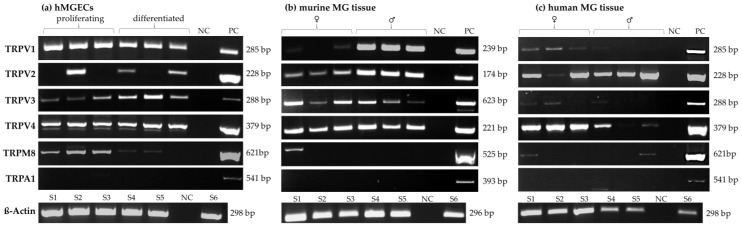
Reverse transcriptase polymerase chain reactions (RT-PCRs) show the gene expression of temperature-sensitive TRPs in the human and murine meibomian gland. (**a**) RT-PCR results in three samples from proliferating and differentiated hMGECs. (**b**) RT-PCR results of murine meibomian gland tissue (three female and three male C57BL; six animals). (**c**) RT-PCR results of human meibomian gland tissue gained from body donors (three female and three male donors). NC = negative control, PC = positive control.

The gene expression of *TRPV1*, *-2*, *-3* and *-4*, *TRPM8* and *TRPA1* was analyzed using RT-PCR in human (three males, three females) and murine (three males, three females) MGs, as well as in immortalized human meibomian gland epithelial cells (hMGECs) (Figure 1). RT-PCR analysis showed the gene expression of *TRPV1* (285 bp), *TRPV2* (228 bp), *TRPV3* (288 bp), *TRPV4* (379 bp) and *TRPM8* (621 bp) in three samples of both proliferating and differentiated hMGEC cultures (Figure 1a). No gene expression could be detected for *TRPA1* in hMGECs. The semi-quantitative analysis for the *TRPM8* PCR signal was much stronger in the proliferating hMGECs than it was in the differentiated hMGECs. RT-PCRs with murine MG tissue (Figure 1b) showed a much stronger signal for *TRPV1* (239 bp) in the male mice than in the female mice. For *TRPV2* (174 bp), *TRPV3* (623 bp) and *TRPV4* (221 bp), the PCRs showed products with comparable signal strengths for all the samples. The PCR for *TRPM8* (525 bp) yielded only one signal for one of the female mice, but no signals in the male mice. Our RT-PCR results showed no gene expression of *TRPA1* (393 bp) in any of the analyzed murine MGs. The RT-PCR in human MG tissue in three samples of both female and male tissue showed a comparatively weak signal for *TRPV1* in the female samples, while only one of the male samples showed any signal at all. *TRPV2* yielded strong signals in all but one female sample, while *TRPV3* only showed weak results in all female samples. The signal in the female samples for *TRPV4* was stronger than the one in the male samples. Notably, *TRPM8* showed signals for only two samples, one female and one male, and there was no sign of *TRPA1* gene expression (Figure 1c). A ß-actin PCR was performed for every utilized cDNA and yielded strong signals for all the samples.

**Figure 2:**
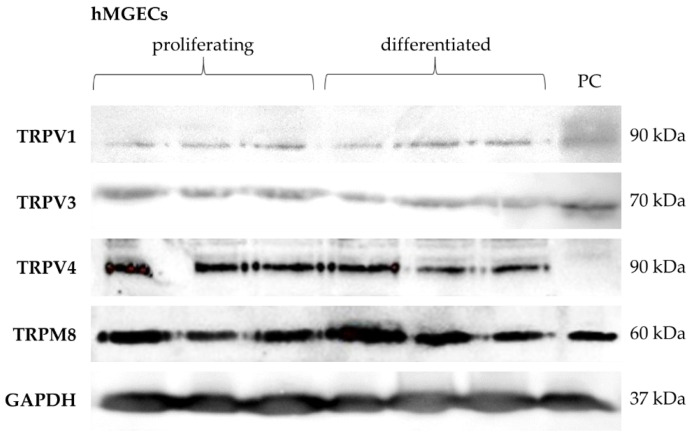
Western blot results show the protein expression of thermosensitive TRPs in cultivated hMGECs. Western blot was performed on three proliferating and three differentiated hMGEC samples. PC = positive control.

Western blot analysis was performed for TRPV1, TRPV3, TRPV4 and TRPM8 in three replicates of proliferating and differentiated hMGECs to verify the gene expression results on the protein level. Our results show the expression of each of these channels at the protein level (Figure 2). The TRPV1 antibody detected a band at the anticipated height of 90 kDa in proliferating and differentiated hMGECs. The band for TRPV3 was detected at approximately 70 kDa. The signal for TRPV4 expression was detected at 90 kDa and the signal for TRPM8 at 60 kDa. For each of the Western blot analyses, a GAPDH antibody was used on the same membrane as an internal positive control, which detected a band at 37 kDa to confirm the integrity and stability of the protein samples. In all Western blot experiments (except for TRPV4) the positive control showed a comparable corresponding band at the same level.

**Figure 3:**
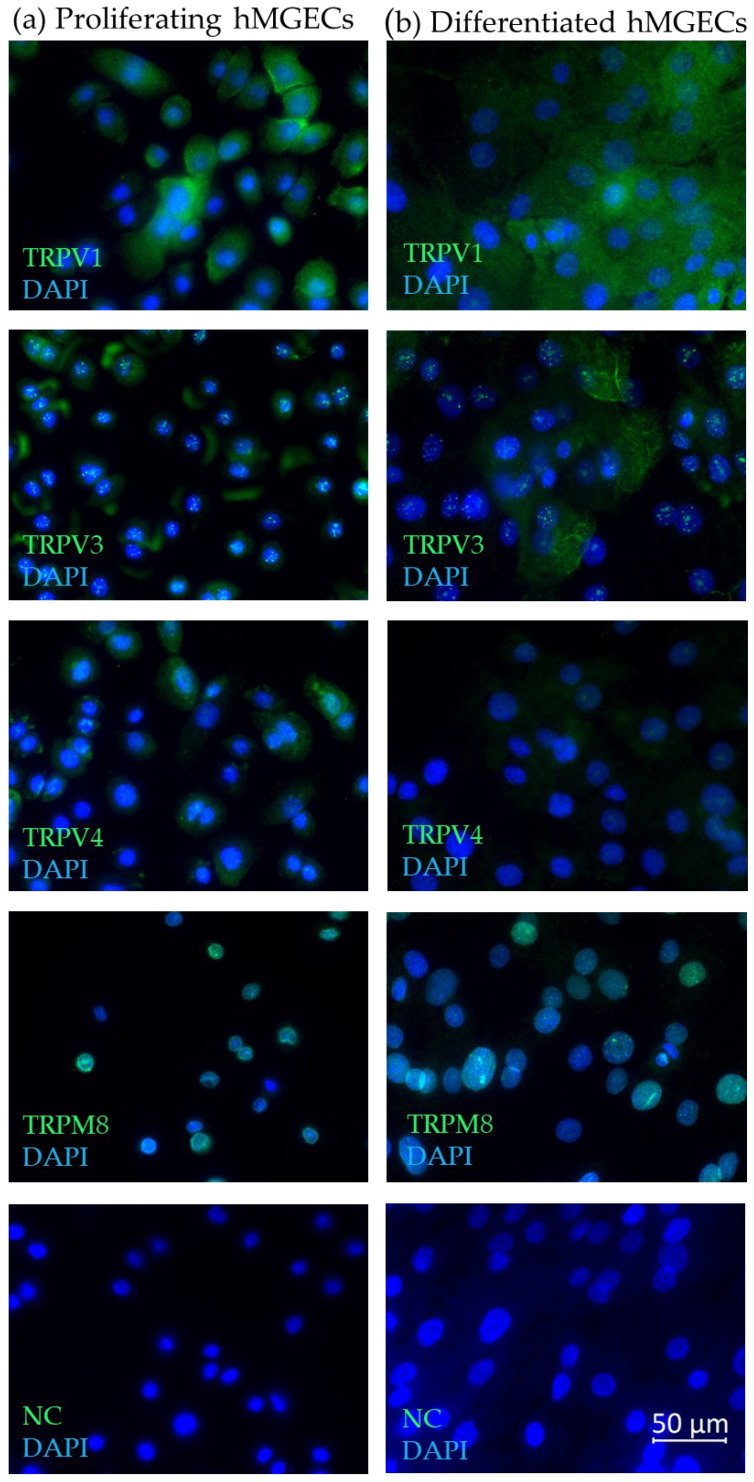
Immunofluorescence results show the expression of TRPV1, TRPV3, TRPV4 and TRPM8 in hMGECs. (**a**) Proliferating hMGECs, (**b**) differentiated hMGECs. The fluorescence microscopic images show an overlay of both the respective antibody (green) and DAPI (blue) signal. All microscopic images are presented in ×600 magnification.

**Figure 4:**
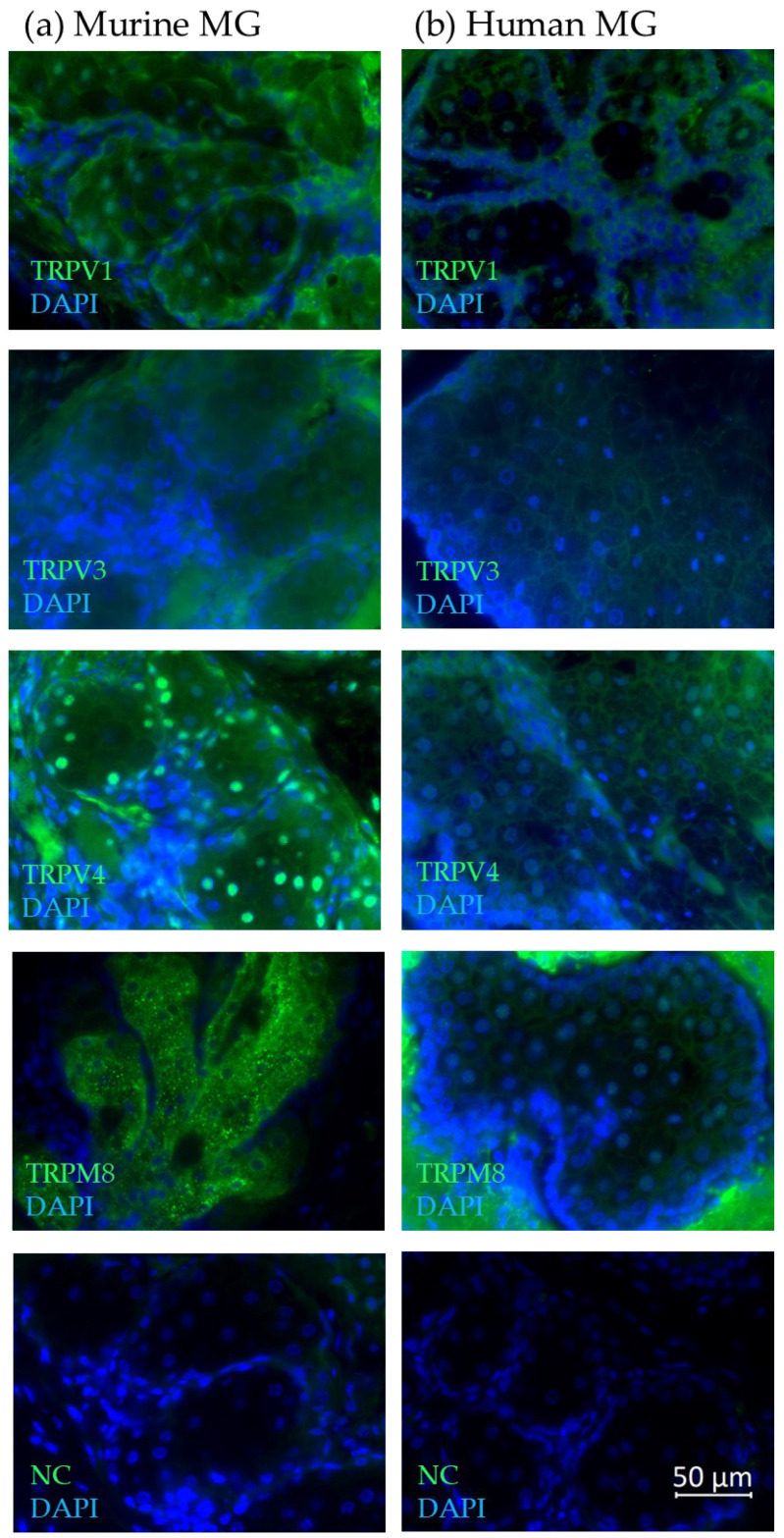
Immunofluorescence results show the expression of TRPV1, TRPV3, TRPV4 and TRPM8 in (**a**) murine and (**b**) human meibomian glands. The fluorescence microscopic images show an overlay of both the respective antibody (green) and DAPI (blue) signal. All microscopic images are presented in ×600 magnification.

Immunofluorescence with antibodies for TRPV1, TRPV3, TRPV4 and TRPM8 confirmed their expression at the protein level and revealed cell- and tissue-specific reactivity in proliferating and differentiated hMGECs (Figure 3a,b), as well as human (Figure 4a) and murine (Figure 4b) MGs obtained from eyelids. The green fluorescence signal corresponds to the cellular- or tissue-specific localization of the corresponding thermo-TRP channel in the examined cells or MG tissues. The blue signal shows the DAPI co-staining against the cell nucleus. In detail, the green fluorescence signal for TRPV1 in both the proliferating and differentiated hMGECs could be located around the cell nucleus in the cytoplasm, with stronger intensity close to the cell membrane in the proliferating hMGECs. The antibody signal for TRPV3 in the proliferating hMGECs could be seen at the edge of the cell, close to the cell membrane and sometimes surrounding the cell nucleus, while in the differentiated hMGECs it stained the cytoplasm, filling the whole cell. Inside the cell nucleus, the antibody signal formed spots of very strong intensity. In the proliferating hMGECs, the signal for TRPV4 was located around the cell nucleus, while the signal in the differentiated hMGECs was much weaker. The TRPM8 signal was limited to the nuclei in both the proliferating and differentiated hMGECs, and no signal could be seen in the cytoplasm or membrane. As expected, the negative controls did not show any specific antibody reaction at the same exposure time.

Furthermore, we examined TRP channel expression in samples of three human and three murine eyelids. In paraffin sections of six C57BL eyelids, the TRPV1 antibody signal in the murine MGs was located both in the cytoplasm and with stronger intensity in some of the nuclei, whereas in the human MGs it was mostly located in the nuclei and the cell membrane with a stronger intensity in the basal cells of the meibomian gland acini (Figure 4a, b). For TRPV3, the fluorescence signal in the murine MGs could be observed in the cytoplasm, leaving out the cell nucleus. In the human MGs, the signal was strongest in the cell membrane in all differentiation stages of meibocytes and weaker in the cytoplasm. In the murine MGs, the strongest fluorescence signal for TRPV4 could be observed in the nuclei and epithelial cells, with the signal becoming weaker in the mature or the hypermature differentiation stage of MG acini. Some fluorescence of the surrounding tissue could be observed. In the human MG, a strong TRPV4 fluorescence signal was located in the cell membrane of meibocytes. In the cytoplasm, the TRPV4 fluorescence signal gave the impression of forming a web. In the mouse MGs, the TRPM8 signal was exclusively in the cytoplasm, excluding the nuclei, whereas in the human MG staining, a very strong non-specific fluorescent signal of the surrounding tissue and a weaker TRPM8-specific signal was found mainly in the meibocytes, the cell membrane and the nuclei, but not in the cytoplasm.

### 2.2. Whole-Cell Current Increase via TRPV1 and TRPM8 in hMGECs

**Figure 5:**
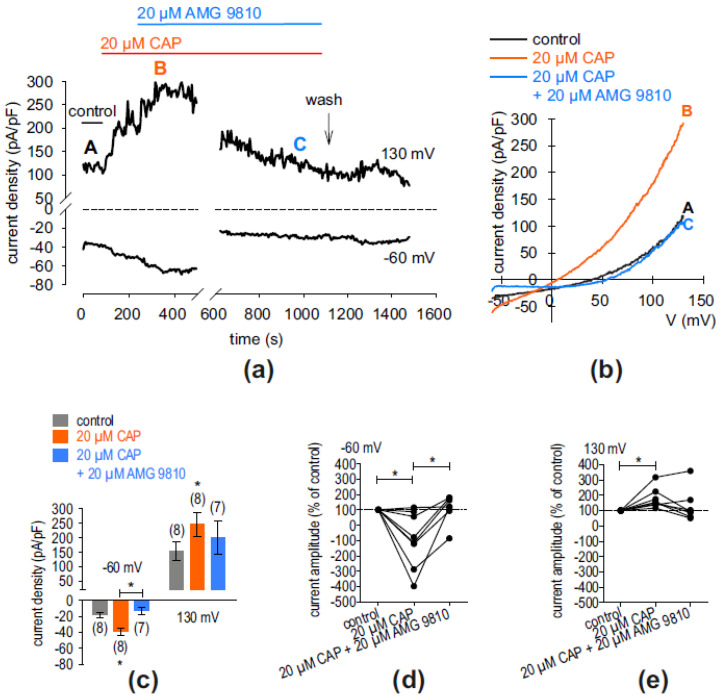
AMG9810 suppressed CAP-induced whole-cell currents in hMGECs. (**a**) Time course recording of whole-cell currents at −60 mV (lower trace) and 130 mV (upper trace). The dashed line is the reference line at 0. The currents were normalized to cell membrane capacitance (current density; pA/pF). An application of 20 µM CAP led to current increases, whereas application of 20 µM AMG9810 suppressed these rises with a delay. Wash out of the blocker induced a small delayed Ca^2+^ transient. (**b**) Original traces of current responses to voltage ramps shown before application (black curve), as well as during application of 20 µM CAP (orange curve, labeled as B) and after adding 20 µM AMG9810 (blue curve, labeled as C). (**c**) Statistical analysis of the patch-clamp recordings of CAP-induced current increases with and without AMG9820. The asterisks (*) designate statistically significant differences of CAP-induced increases in inward whole-cell currents compared to controls (*n* = 7–8, * *p* < 0.05; paired tested). (**d**) Maximal inward current amplitudes induced using a voltage step from 0 mV to −60 mV are shown as a percentage of control values before application of 20 µM CAP (control set to 100%; dashed line) (*n* = 8, * *p* < 0.05; one sample *t*-test; *n* = 7, * *p* < 0.05, paired test. (**e**) Same analysis as shown in (**d**) but concerning the outward currents at a voltage step from 0 mV to 130 mV. The CAP-induced outward currents increased (*n* = 8, * *p* < 0.05; one sample *t*-test).

The characteristics of cells are dependent on calcium-dependent cellular mechanisms. Intracellular calcium is substantially regulated via TRPs. Therefore, in this set of experiments, whole-cell currents were measured after the application of CAP (20 µM) with and without the specific TRPV1 blocker AMG9820 (10 µM) for the identification of functional TRPV1 channels in cultivated hMGECs. Similarly, 20 µM icilin was added with or without the specific TRPM8 blocker AMTB (10 µM) for TRPM8 identification. Figure 5a shows that an extracellular application of 20 µM CAP led to an increase in whole-cell out- and inward currents. Specifically, the latter increased from −18.31 ± 3.45 pA/pF (control) to −39.29 ± 4.31 pA/pF (both *n* = 8; * *p* < 0.05) (Figure 5c). This current increase could be suppressed in the presence of 10 µM AMTB to 13.03 ± 4.35 pA/pF) (* *p* < 0.05; *n* = 7) (Figure 5c). Figure 5b shows the current voltage relationship of whole-cell currents in control conditions (black trace) and in the presence of 20 µM CAP without AMG9810 (orange trace) and with AMG9810 (20 µM) (blue trace). Figure 5d,e show normalized current amplitudes (% of control) of CAP-increased inward- and outward currents with and without AMG9810. In the presence of CAP, the inward currents increased from 100% (set as control) to −93 ± 64% (*n* = 8; * *p* < 0.05). In the presence of 20 µM AMG9810, these currents decreased to 110 ± 35% (*n* = 7; * *p* < 0.05). Similar results could be registered concerning the outward currents (*n* = 8; * *p* < 0.05). However, an inhibitory effect could not be detected in every cell and did not reach statistical significance.

**Figure 6:**
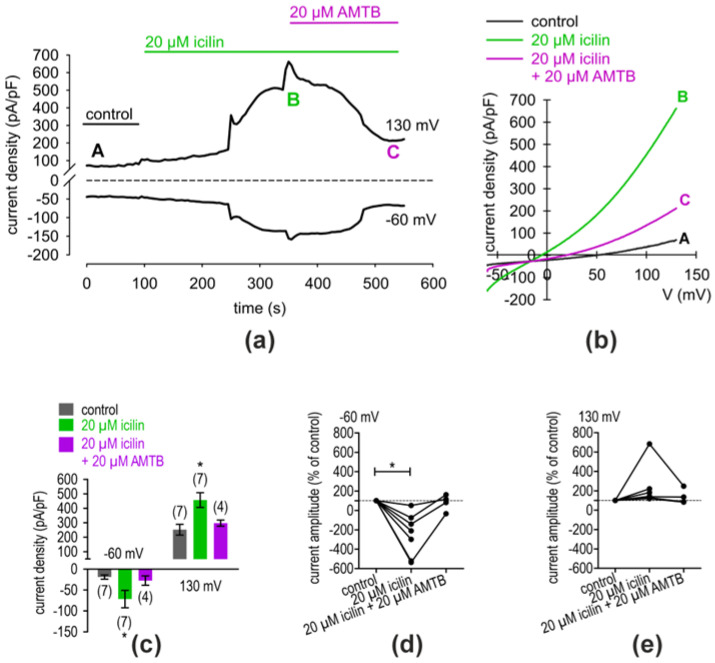
AMTB suppressed icilin-induced whole-cell currents in hMGECs. (**a**) Time course recording of whole-cell currents at −60 mV (lower trace) and 130 mV (upper trace). The dashed line is the reference line at 0. The currents were normalized to cell membrane capacitance (current density; pA/pF). An application of 20 µM icilin led to current increases, whereas application of 20 µM AMTB suppressed these rises with a delay. (**b**) Original traces of current responses to voltage ramps shown before application (black curve), as well as during application of 20 µM icilin (green curve, labeled as B), and after adding 20 µM AMTB (violet curve, labeled as C). (**c**) Statistical analysis of the patch-clamp recordings of icilin-induced current increases with and without AMTB. The asterisks (*) designate statistically significant differences of icilin-induced increases in inward whole-cell currents compared to controls (*n* = 4–7, * *p* < 0.05; paired tested). (**d**) Maximal inward current amplitudes induced using a voltage step from 0 mV to −60 mV are shown as a percentage of control values before application of 20 µM icilin (control set to 100%; dashed line). The icilin-induced outward currents increased (*n* = 7, * *p* < 0.05; one sample *t*-test). (**e**) Same analysis as shown in (**d**) but concerning the outward currents at a voltage step from 0 mV to 130 mV.

Similar experiments were carried out regarding the identification of functional TRPM8 expression, which are summarized in Figure 6. Figure 6a shows the time course of the experiment whereas Figure 6b shows the corresponding current voltage relationship at the time points marked with the capitalized letters. At this point, icilin and AMTB were used (both 20 µM). As a result, icilin increased the whole-cell inward currents from −18.83 ± 5.02 pA/pF (control; *n* = 7) to −71.54 ± 20.82 pA/pF (* *p* < 0.05; *n* = 8) (Figure 6c). Similar results could be registered concerning the outward currents (*n* = 7; * *p* < 0.05) (Figure 6c). An inhibitory effect could be observed but did not reach statistical significance. Figure 6d,e summarize an analysis at which the control currents were set to 100%. After the application of 20 µM icilin, the inward currents increased from 100% to −248 ± 84 % of the controls (*n* = 7; * *p* < 0.05). In summary, the patch-clamp data analyses indicated that TRPV1 and TRPM8 are functionally expressed in hMGECs.

### 2.3. Calcium Regulation via TRPV1 and TRPM8 in hMGECs

**Figure 7:**
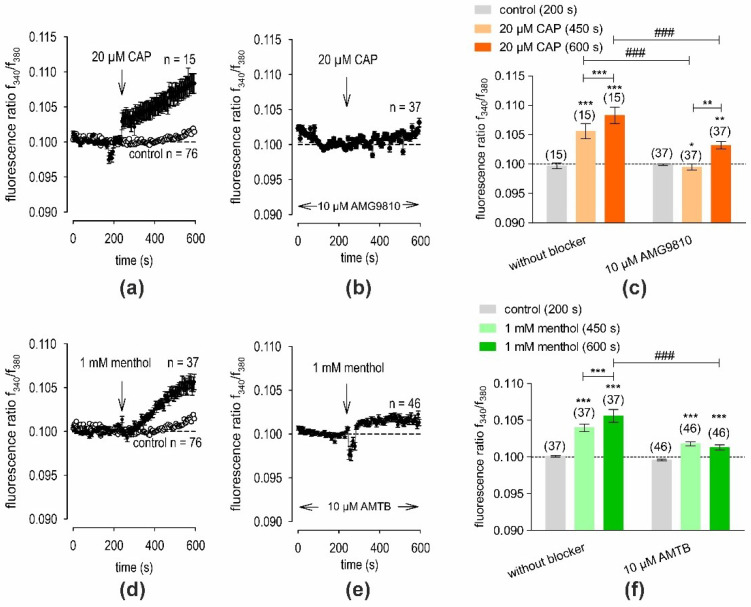
CAP or menthol increased intracellular Ca^2+^ [Ca^2+^]_i_ in fura-2-loaded hMGECs. Time-dependent Ca^2+^ changes are shown as fluorescence ratios (f340/f380), which are proportional to the [Ca^2+^]_i_ levels in hMGECs. Data are represented as mean ± SEM. n indicates the number of cells measured in this set of experiments. Dashed line represents the reference line for baseline value (0.1). Arrows mark the time (240 s) at which the agonist was added. (**a**) Application of 20 µM CAP increased [Ca^2+^]_i_ (filled circles; (*n* = 15). For the control without CAP application, no changes in the [Ca^2+^]_i_ level could be observed (*n* = 76) (open circles). (**b**) The effect of CAP could be suppressed in the presence of the specific TRPV1 blocker AMG9810 (10 µM; *n* = 37). (**c**) Summary of the experiments with CAP and AMG9810. Bars represent mean values ± SEM of the fluorescence ratio at 200 (control), 450 and 600 s (CAP). The numbers of cells measured in both experiments are indicated in brackets above the bars. The asterisks (*) indicate statistically significant differences with and without CAP (*n* = 15 to *n* = 37; *** *p* < 0.001, ** *p* < 0.01, * *p* < 0.05; paired tested). The hashtags (#) refer to unpaired data with and without AMG9810 (### *p* < 0.001). (**d**–**f**): Same experiments as shown in (**a**–**c**). Instead of CAP 1, mM menthol was used (filled circles; *n* = 37) and instead of 10 µM AMG9810, 10 µM of TRPM8 blocker AMTB was added (*n* = 46).

To assess TRPV1 function in hMGECs, the effects of the TRPV1 agonist CAP (20 µM) on intracellular calcium [Ca^2+^]_i_ levels were measured. Before applying 20 µM CAP, baseline measurements were conducted using fura-2/AM dye to obtain a control measurement. Ringer-like solution (RLS) was applied for the initial 4 min of the measurements (control). The baseline f340 nm/f380 nm ratio was 0.09862 ± 0.00048 (*n* = 15) at 200 s. Subsequently, an extracellular application of 20 µM CAP was performed at 240 s (indicated by an arrow in Figure 7a). The f340 nm/f380 nm ratio increased to 0.1083 ± 0.0014 at 600 s (*n* = 15, *** *p* < 0.001), indicating an overall increase in the [Ca^2+^]_i_ concentration (Figure 7a). To investigate whether the increase in the Ca^2+^ influx in the hMGECs was due to CAP-induced TRPV1 activation, a negative control experiment was conducted in the presence of the selective TRPV1 antagonist AMG9810 (10 µM). Cells were pre-incubated with 10 µM AMG9810 and fura-2/AM simultaneously for 20–40 min. Accordingly, a visible blocking effect was observed (Figure 7b). Notably, the increase in Ca^2+^ was abolished by the administration of 10 µM AMG9810. Specifically, it decreased to 0.1032 ± 0.0006 at t = 600 s (*n* = 37, ### *p* < 0.001) (Figure 7c). These findings collectively suggest the functional expression of TRPV1 in hMGECs, as evidenced by the identification of Ca^2+^ changes documented using CAP and AMG9810.

The same experimental design was used to assess TRPM8 function in hMGECs. This time, the effect of the selective TRPM8 agonist, menthol (1 mM), on [Ca^2+^]_i_ levels was measured. Before applying 1 mM menthol, baseline measurements were conducted which had an f340 nm/f380 nm ratio of 0.1001 ± 0.0001, with *n* = 37 at 200 s. Subsequently, the extracellular application of 1 mM menthol led to an increase in the f340 nm/f380 nm ratio to 0.1056 ± 0.0009 at t = 600 s (*n* = 37, *** *p* < 0.001), indicating an overall increase in the [Ca^2+^]_i_ concentration (Figure 7d). As with the CAP experiments, a negative control experiment was conducted in the presence of the selective TRPM8 antagonist AMTB (10 µM). As shown in Figure 7e, the increase in [Ca^2+^]_i_ was clearly suppressed by 10 µM AMTB. Accordingly, the f340 nm/f380 nm ratio decreased to 0.1013 ± 0.0004 at t = 600 s (*n* = 46, ### *p* < 0.001). The results of the two-test series are summarized in Figure 7f. Therefore, the functional expression of TRPM8 in hMGECs was suggested, as shown by the intracellular Ca^2+^ analysis using menthol and AMTB. Taken together, TRPV1 and TRPM8 channels in hMGECs could be detected for the first time.

### 2.4. Influence of TRPV1 and TRPM8 Activation on hMGEC Lipogenesis

**Figure 8:**
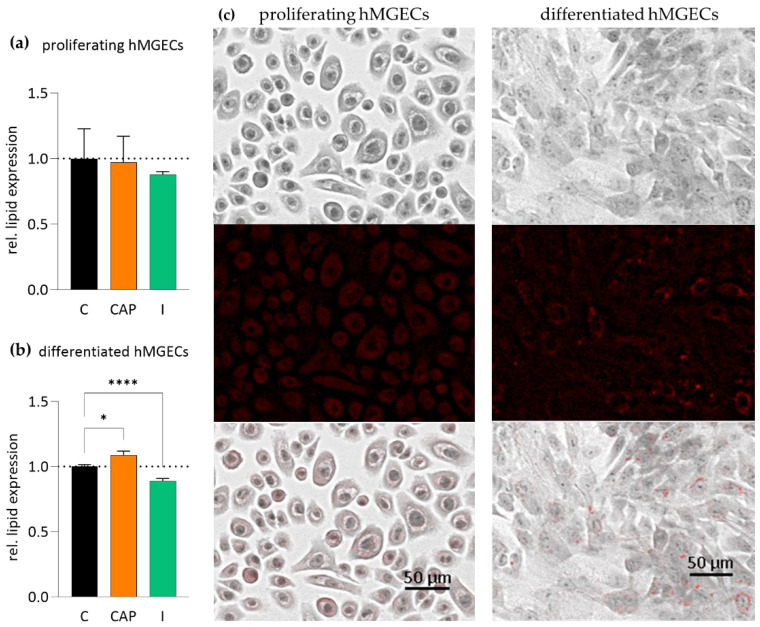
Results of oil red O staining (ORO) after TRPV1 and TRPM8 stimulation in proliferating and differentiated hMGECs. hMGECs were stimulated for 24 h with CAP (10 µM) or icilin (I, 100 µM). The lipid accumulation in the cells was stained with ORO, documented photographically and analyzed using ImageJ software (version 1.54 h). The bars represent the ratio of the lipid accumulation compared to untreated controls. (**a**) In the proliferating hMGECs, CAP and icilin showed no significant impact on lipid accumulation after 24 h. (**b**) In the differentiated hMGECs, there was a significant increase in lipid content after TRPV1 activation and a decrease after TRPM8 activation. (**c**) Representative images of the experiments summarized in (**a**,**b**). The pictures show the stained proliferating and differentiated hMGECs in ×600 magnification, the bright field image, the ORO staining (red) and an overlay of both pictures. Asterisks (* *p* < 0.05, **** *p* < 0.0001; unpaired, ordinary one-way ANOVA test indicates statistically significant results for the stimulation of TRPV1 and TRPM8).

Additionally, we analyzed the impact of the TRPV1 agonist CAP (10 µM) and the TRPM8 agonist icilin (100 µM) on the lipogenesis in cultivated hMGECs using oil red O (ORO) staining. The treatment of proliferating hMGECs with 10 µM CAP for 24 h showed no significant impact on lipid expression compared to a control. The icilin-treated proliferating hMGECs showed a more visible decrease but this did not reach statistical significance (Figure 8a). The differentiated hMGECs treated with CAP showed an increase in their lipid accumulation by 8.8 ± 3.02% (*n* = 12; *p* < 0.05) (Figure 8b, Table 1). In contrast, the treatment with icilin led to a significant decrease in lipid accumulation of 10.97 ± 1.73% (*n* = 12; **** *p* < 0.0001) (Figure 8b, Table 1).

## 3. Discussion

### 3.1. Temperature-Sensitive TRPs Are Expressed and Functionally Active in the Meibomian Glands

Our results demonstrate for the first time that thermosensitive TRPs, such as TRPV1, -2, -3 and -4, as well as TRPM8, are expressed in the MGs of mice and humans, as well as in the immortalized human meibomian gland epithelial cell line (hMGEC). In addition, we could show that TRPV1 and TRPM8 are functionally active in cultivated hMGECs in vitro.

Our expression analysis results showed the gene and protein expression for TRPV1, -2, -3 and -4, and TRPM8 in the analyzed MGs and hMGECs (Figure 1, Figure 2 and Figure 3). The RT-PCRs for the human cadaver samples yielded varying results, which could be explained by the different stages and times of decomposition the cadavers had undergone before preservation. Interestingly, *TRPM8* showed weaker fluorescence signals for the differentiated hMGECs than for the proliferating stage, which could identify it as a possible marker of differentiation in this established cell culture model. This possibility requires further investigation. *TRPV1* in the murine MG tissue showed hardly any signal in the female mice and a very strong signal in the male mice. This is possibly a gender-specific difference, yet the same could not be observed in the human tissue. However, the number of animals examined was too limited to support a final conclusion. On the other hand, no differences in TRP sex-specific expression are known. Therefore, a further investigation into the issue could lead to interesting new approaches, especially when considering sex-specific or even personalized approaches in medicine or pharmacology. The expression in MGs was consistent with the presence of temperature-sensitive TRPs in other ocular tissues including our previous studies at the ocular surface [21,34,35,36,37]. The only exception and unexpected result was the lack of gene expression of *TRPA1*. *TRPA1* is not only a contributor to pain and inflammation in general but it was also found to play a role in DED in a rat model, where the stimulation of TRPA1 led to a significant increase in eye wiping and blinking [39,40]. The lack of *TRPA1* expression we found in the MGs is surprising considering the cold receptor can be found in the cornea, conjunctiva and other ocular tissues [41]. As with other thermo-TRPs, there have been attempts to develop TRPA1-targeting pain relievers in the form of eyedrops, among others [42]. However, we found no *TRPA1* gene expression in murine or human MGs or hMGECs. This lack of expression could be investigated in further studies and confirmed at the protein level or with functional experiments. In addition, the expression of *TRPV2* was also less certain because we only examined it at the gene level but not at the protein level. The RT-PCR results for *TRPV1*, *TRPV3*, *TRPV4* and *TRPM8* were confirmed at the protein level using Western blot and immunofluorescence analyses (Figure 2 and Figure 3). The Western blot results showed a clear expression of the investigated channels in the immortalized cell line, as did the immunofluorescence. Here, the investigation was not limited to the cell line but was also confirmed in murine and human tissue. A slight fluorescence of the surrounding tissue could be observed in some cases but the comparison with the negative control showed a clear and specific fluorescence signal in MG tissue. The commercial antibodies used in our IF investigations underwent a multi-stage validation process and had a high specificity, validated in the WB analyses shown in Figure 2. 

Consistent with the expression results, we limited the present functional experiments to the following two most researched and well-known thermo-TRPs: TRPV1, the heat and capsaicin receptor; and TRPM8, the cold and menthol receptor. The functionality of TRPV1 and TRPM8 were shown in the planar patch-clamp recordings (Figure 5 and Figure 6) and in the fura-2 calcium measurements (Figure 7). The resulting changes in the membrane whole-cell currents of hMGECs during the planar patch-clamp evaluation and the treatment with CAP and AMG9810 [43,44] for TRPV1, as well as menthol/icilin and AMTB [45,46,47], validated the functional expression of TRPV1 and TRPM8. Similar current response patterns could be observed in human corneal epithelial cells [48]. While CAP is a very potent and specific agonist for TRPV1, icilin activates not only TRPM8 but also TRPA1 [49]. Notably, none of the performed RT-PCRs showed any signs of *TRPA1* gene expression, neither in cells nor tissues. Therefore, we conclude that TRPA1 is not expressed in the MGs. Accordingly, icilin was used as a selective TRPM8 agonist in this gene expression pattern [47]. The chosen antagonists AMG9810 and AMTB are very selective and led to a decrease in membrane currents in both cases, confirming that TRPV1 and TRPM8 are active in the hMGECs. This is consistent with findings in other ocular tissues such as cornea, conjunctiva, uveal melanoma and retinoblastoma cells [21,34,35,36,37].

### 3.2. TRPV1 and TRPM8 Activation Influence the Lipid Synthesis of Meibocytes

TRPs are mostly discussed in a neuronal/sensory-physiological context but they are also functionally expressed and play important roles in non-neuronal cells and tissues. They are involved in many physiological processes throughout the human body such as apoptosis, cell growth and migration, angiogenesis, muscle contraction and many more. There are also over 50 known lipid molecules that can modulate TRP activity [50,51] and there are examples of TRP activation influencing secretory functions of epithelial cells [52], so the question of whether the channels have an influence on the lipid synthesis and/or accumulation in hMGECs had to be raised. Our oil red O results showed the stimulation of TRPV1 with 10 µM CAP, leading to a statistically significant increase of 9% in the lipid accumulation of the hMGECs. This is particularly interesting as there are similar experiments on cultivated sebaceous cells in which the treatment with CAP inhibited lipid production in SZ95 sebocytes [53]. In these experiments, a trend could be observed, showing a stronger inhibition with higher concentrations of CAP, the highest concentration being the 10 µM we used in our stimulations. The meibomian gland is a specialized sebaceous gland so it is surprising that the activation of TRPV1 had different effects on the cells. In contrast, our stimulation of TRPM8 with icilin led to a statistically significant decrease of 11% in lipid accumulation (Figure 8). These findings are particularly interesting because the addition of heat, e.g., by means of a warming mask, improves the meibum melting point, which is altered in MGD [54]. It could be shown that the anti-evaporative effect of the tear film is based in particular on the wax esters of the meibum at a given temperature [55]. Against this background, the participation of TRPV1 and TRPM8 in the lipid synthesis of the meibocytes could have significant implications for TRP-targeting drugs at the ocular surface in the treatment of MGD and, consequently, for dry eye disease (DED). 

At this point, we want to add that our experiments were performed on body donor tissue and a cultivated immortalized cell line which results in certain limitations. Therefore, the differences in thermo-TRP channel expression we found, particularly in our RT-PCR results, could be attributed to the duration between death and tissue removal, (unknown) pre-existing conditions or the medication of body donors. While experiments in vitro can give us a general idea of the body’s molecular structure and workings, they do not reflect the actual conditions in the living body one-to-one and should always be confirmed using follow-up studies with tissue from other sources. 

DED is closely linked with TRPs. There have been countless investigations into their involvement and possible therapeutic application for this disease [14,15,16,17,18]. The inflammation of the ocular surface causes an overactivation of the cold thermoreceptors on the trigeminal nerve endings leading to a sensation of dryness and pain [56], while TRPV1 has been identified as a key player in the transduction of heat, irritation and pain signaling, thereby mediating DED symptoms induced by hyperosmolarity [14]. Furthermore, TRP channelopathies are linked to renal, skeletal, cardiovascular and nervous system pathologies. Their involvement in diseases of the respiratory tract, inflammatory bowel disease, bladder control, cancer, diabetes and many more has been shown in various studies [15,16,17]. Ion channel drugs are a multi-billion dollar market and TRPs make very promising drug targets [57]. Since both their activation and deactivation can be beneficial, and they are expressed ubiquitously in the human body, a systemic application has proven problematic. Drugs in phase 1 or 2 trials have been pulled because of unforeseen side effects such as hyperthermia or noxious heat perception [16,58]. However, a local/topical application such as in eye drops could still be possible and seems very promising. TRPs have been identified as possible drug targets for improving DED years ago [14,18,19]. According to Fakih et al., “drug targeting TRPs may be of therapeutic benefit in the clinical setting of ocular pain” because of their involvement in hyperalgesia and the mediation of DED symptoms but also their direct influence on basal tear production [14,59]. The question of whether a TRPM8 agonist or an antagonist is more beneficial to the treatment of DED still lacks a definitive answer. Both have been tested and have shown advantages and disadvantages. TRPM8 agonists, such as menthol, led to a cooling sensation and increased lacrimation in a DED mice model. However, depending on the dosage, they also led to increased nocifensive behavior. Selective TRPM8 antagonists, such as AMTB or BCTC, decrease the hypertonic saline-evoked orbicularis oculi muscle activity and corneal drying [18]. Selective TRPV1 antagonists have been shown to inhibit the upregulation of genes involved in inflammatory and neuropathic pain [18]. Several TRP channel modulating drugs are currently being tested in clinical trials [60,61,62,63,64], mostly aiming for a downregulation of the ocular surface pain caused by DED. If these drugs should go further in their trials, it is important to know what kind of effect they might have on adjacent tissues of the ocular surface and lacrimal apparatus. One of these drugs currently being tested is the topical TRPM8 agonist, cryosim-3 (C3), which is applied on the eyelid margins with a cotton swab [60,61]. While resulting in a short-term feeling of pain relief and cooling, the localization of this agent is predestined for affecting the MGs. According to our findings it could lead to a decreased lipid accumulation in meibocytes, possibly worsening the underlying condition causing the symptoms. The same goes for another TRPM8 agonist, AR-15512 [62], which has already entered a phase 3 clinical trial, (NCT05493111), possibly running into the same risks. A promising TRPV1 targeting drug is the siRNA SYL1001, or Tivanisiran, a specific TRPV1 inhibitor. It has successfully passed two phase 2 trials and has now entered a phase 3 trial, NCT05310422. The results show a significant reduction in ocular pain and hyperemia, and possibly even an improvement in tear quality [63,64]. Since TRPV1 is known to be of high importance in the transmission of pain and inflammation [65], this does not come as a surprise. However, a closer look at the influence of TRPV1 inhibition on the MGs might be warranted. Our results showed an increase in lipid accumulation through TRPV1 activation (Figure 8a), which does not automatically suggest the opposite for TRPV1 inactivation, but further investigation of the issue seems justified. 

In summary, it is important to investigate the relationship between the activation of thermo-TRPs and lipid accumulation in MGs in further studies. The testing of novel thermo-TRP agonists and antagonists, if possible in suitable in vivo animal models instead of cell lines, seems particularly useful here. Our results showed for the first time the functional expression and activity of individual thermo-TRPs in MGs. These results could be relevant in a clinical context, as thermal TRP activation also influences meibocyte lipogenesis in hMGECs and may have an influence on MGD or DED, possibly serving as an important approach for the development of a pharmacological treatment option. 

## 4. Materials and Methods

### 4.1. Cells

An immortalized human meibomian gland epithelial cell line (hMGECs, kindly provided by David Sullivan, Schepens Eye Research Institute, Boston, MA, USA) was cultivated at 37 °C, 5% CO_2_ under two different conditions as described previously [66]. First, for proliferation in serum-free medium (SFM, Gibco #17005-034 Thermo Fisher Scientific, Waltham, Massachusetts, USA, 2.5 µg EGF, 25 mg BPE) and for differentiation, the cells were incubated in in 10% fetal calf serum containing medium (DMEM F-12 HAMS, Biochrom/Merck, Darmstadt, Germany, #FG4815, EGF) for 24 h [67]. Medium was changed every two days during cultivation. 

### 4.2. Tissues

Human eyelids were obtained from human cadavers donated by written testamentary disposition and in accordance with German law to the Institute for Functional and Clinical Anatomy at the Friedrich-Alexander-University Erlangen-Nürnberg (FAU). The dissection of the bodies took place 4–12 h after death. The donors suffered no diseases involving or affecting the lacrimal apparatus or had any kind of eye trauma. 

Murine tissue was obtained from C57/BL6 mice, aged 18–22 weeks. All experiments were conducted in accordance with the ARVO Statement for the use of animals in ophthalmic and vision research and the national ethical committee for animal experimentation (FELASA) and were approved and registered by the Animal Welfare Office of Friedrich-Alexander University (TS-12/14 Anatomie II, 26 August 2020). 

The tissues were prepared for paraffin embedding and fixation in 4% paraformaldehyde. If used for molecular biology experiments the tissues were immediately frozen at −80 °C.

### 4.3. RNA Isolation and cDNA Synthesis

The eyelids of three female and three male human cadavers (aged 58–89 years), as well as three female and three male mice were dissected under a microscope until only the meibomian gland tissue was left. The tissues were then sliced into small pieces and placed into Lysis tubes containing 400 µL of RNA Solv (SKU: R6830-02, Omega Bio-Tek, Norcross, Georgia, USA), followed by submerging the tubes in liquid nitrogen. Homogenization was carried out in a Speedmill Plus (Analytik Jena AG, Jena, Germany). Centrifugation was used to separate the insoluble material (12,000× *g*, 5 min, 4 °C). Total RNA was achieved using GenElute Kit (Sigma Aldrich, Merck) according to the manufacturer’s protocol. Isopropanol and repeated ethanol precipitation was used to purify the crude RNA and an RNAse-free DNAse I (37 °C, 30 min, ThermoFisher Scientific, Waltham, MA, USA) was used to digest any contaminating genomic DNA. The DNAse reaction was stopped at 65 °C for 10 min. The generated total RNA was used to generate sample cDNA with the RevertAidTM Reverse Transkriptase-Kit (ThermoFisher Scientific, Waltham, MA, USA) according to the manufacturer’s instructions. For each reaction, 4 µg of RNA and 2 µL of 10 pmol Oligo (dT) primers were used. The resulting cDNA was stored at −20 °C.

### 4.4. Reverse Transcriptase Polymerase Chain Reaction (RT-PCR)

For RT-PCR the Roth Kit was used according to the manufacturer’s instructions. To verify the integrity and stability of each transcribed cDNA an ß-actin PCR was performed. The protocol for the gene-specific PCR included 1 µL cDNA, 12.5 µL Roti-Pol Hot-TaqS Red Mix (#9256), 10.5 µL RNAse free H_2_O and 0.5 µL of both the forward and reverse primers (Table 1) per sample. The expression of each gene was investigated using specifically designed intron-spanning primer pairs. Base pair values were equivalent to the expected amplification products compared with GenBank data. A negative control with aqua dest (a.d.) instead of cDNA was performed to confirm the specific amplification of only cDNA and a positive control based on an extensive literature search confirmed the functionality of the designed primers (Table 2).

### 4.5. Quantitative Real-Time Polymerase Chain Reaction (qPCR)

The effect of simulated inflammatory and hyperosmolaric conditions (as they occur during DED [7]) on TRPV1 and TRPV4 expression on proliferating and differentiated hMGECs was investigated using qPCR. The cells were stimulated with 10 ng/mL IL1ß or 10 ng/mL TNFα for 6 h and 24 h, as well as with hypotone (medium diluted 1:1 with a.d.) and hypertone medium (concentrations of 450, 500, 550 mOsm NaCl) for 24 h. Takyon MasterMix (Eurogentec, Seraring, Belgium) was used according to manufacturer’s instructions and duplicates of four biological replicates were amplified and analyzed using a LightCyclerR 480 (Roche, Basel, Switzerland). A no-template control served as the negative control. Data were analyzed according to ddCt analysis by Pfaffl [68].

### 4.6. Protein Isolation and Western Blot

For protein isolation hMGECs, human and murine MG tissues were mixed with 300 µL of Triton buffer and homogenized in a Speedmill Plus (Analytik, Jena, Germany) for two minutes. After resting on ice for 30 min they were centrifuged (13,000 rpm, 30 min, 4 °C). The resulting supernatant contained the proteins and was stored at −80 °C. The protein concentration was analyzed by means of the Bradford reagent. For Western blot analysis, 30 µg protein was incubated with 7 µL 5× Loading dye (containing DTT) at 95 °C for 5 min, cooled on ice and centrifuged before being loaded onto a 6% SDS agarose gel. The gel was submerged in Laemmli buffer and the proteins were bundled at 10 mA and 300 mV for 10 min, then separated at 10 mA and 180 V for 40 min. The proteins were transferred from the SDS gel onto a nitrocellulose membrane (GE Healthcare, Chicago, Illinois, USA, #10600008) in a semidry blot system using a Trans-Blot Turbo Transfer System (17001915, Bio Rad, Hercules, CA, USA). The membrane was stained with Ponceau stain for 2 min, washed in a.d. and submerged in a blocking solution (5% milk powder in TBS-T) for 1 h. The antibody was diluted in the blocking solution at the concentration provided by the manufacturer (Table 3), and the membrane was covered in the antibody mix for 1 h at room temperature and at 4 °C overnight. The membrane was washed 3 times for 15 min in TBS-T and incubated with the secondary antibody (Table 3), diluted in the blocking solution at a concentration of 1:5000 for 2 h at room temperature. After washing it 3 times for 15 min in TBS-T, it was covered in ECL mix for 5 min (Millipore, Merck; #WBKIS0500) for chemiluminescence detection and pictures were taken with an iBright CL 1500 (A44114, Thermo Fisher Scientific, Germany). For GAPDH, the membrane was stripped (65 °C, 45 min), washed with TBS-T and suspended in blocking solution for 1 h. A GAPDH antibody (Table 3) diluted in blocking solution was added for 1 h at room temperature and 4 °C overnight. The membrane was washed three times in TBS-T and the secondary antibody was added for 2 h at room temperature. An ECL mix was used for chemiluminescence detection [69]. As the question posed by this project only concerned the general expression of TRPs in the meibomian gland, the Western blot results were not quantified. 

### 4.7. Immunofluorescence

For immunofluorescence (IF) analysis, three human and three murine (upper + lower) eyelids were fixed in paraformaldehyde (PFA), embedded in paraffin and sliced into 5 µm slices. The slices were dewaxed in xylol and a descending ethanol series as described previously [70]. The slides were incubated in citrate buffer (pH = 6) for 10 min at 80 °C and left at room temperature for 3 h. They were washed with a.d. and TBS-T before incubating them with trypsine at 37 °C for 10 min. The slides were then washed three times with TBS-T and blocked in 2 g milk powder in 100 mL PBS with 100 µL Tween 20 for 1 h at room temperature. The slides were washed with TBS-T twice before adding the primary antibody (Table 3) for 1 h at room temperature and 4 °C overnight. The slides were washed with TBS-T three times before adding the secondary antibody (Table 3) for 1 h. After washing the slides three times with TBS-T the cell nuclei were stained with DAPI mix (1:1000, Carl Roth GmbH, Karlsruhe, Germany, 6335.1) for 10 min and then covered for analysis.

Images were acquired using a Keyence BZ800X microscope (Keyence GmbH, Neu-Isenburg, Germany). 

### 4.8. Planar Patch-Clamp Recordings

Whole-cell recordings were obtained using a planar patch-clamp setup (Port-a-Patch; Nanion^®^, Munich, Germany). The PatchMaster software (Version 2x90.5 from HEKA Electronic, 67466 Lamprecht/Pfalz, Germany) was used to record and evaluate whole-cell currents and measuring parameters (Version 2.73 from HEKA Electronic, 67466 Lamprecht/Pfalz, Germany). At first, a single cell suspension was prepared and transferred in an external measuring solution containing (in mmol/L) 140 NaCl, 4 KCl, 1 MgCl_2_, 2 CaCl_2_, 5 D-Glucose monohydrate and 10 HEPES-acid at pH 7.4 and 298 mosmol/L. A 5 µL single cell suspension was checked in the microscope and (with sufficient cell density) subsequently placed on top of a small aperture of a micro-chip. The cell-attached configuration was reached when one single cell contacted the aperture through a negative pressure, supplied by a software-controlled pump (Nanion^®^, Munich, Germany). Before, the chip was filled with 5 µL of internal solution inside the chip (calcium-free internal measuring solution containing the following in mmol/L: 50 CsCl, 10 NaCl, 60 CsF, 20 EGTA and 10 HEPES (pH ≈ 7.2 and ≈ 288 mosmol/L)). Extracellular solution was manually applied onto the chip using a small pipette. Mean membrane capacitance (12.33 ± 1.42 pF; *n* = 15) and mean access resistance (18.93 ± 2.98 MΩ; *n* = 15) were determined using the PatchMaster software. Series resistances, fast and slow capacitance transients, as well as leak currents, were compensated using the patch-clamp amplifier (HEKA Electronic, 67466 Lamprecht/Pfalz, Germany). Liquid junction potential was determined and considered after Barry [71]. All experiments were performed at ≈23 °C room temperature unless stated otherwise. The holding potential (HP) was set to 0 mV to eliminate any possible contribution of voltage-dependent Ca^2+^-channel activity. Every 5 s, a voltage ramp stimulation without steps (voltage change from −60 to +130 mV) for 500 ms was applied. Current recordings were leak-subtracted and cells with leak currents above 100 pA were discarded from data analysis. Notably, the hMGECs are small fragile cells and difficult to patch. A limitation of these measurements was that leak currents and access resistance partially fluctuated during the recordings. In many cases, the recordings had to be briefly interrupted for a new compensation (e.g., see Figure 5a). Currents were normalized with respect to cell membrane capacitance to obtain current density plots (pA/pF). 

### 4.9. Calcium Fluorescence Imaging 

To conduct cellular measurements, hMGECs were seeded at a 1:4 dilution on 15 mm glass coverslips in 12-well plates. After 24 h, 1 µM of fura-2/AM fluorescent dye was added to the coverslips, which were placed in to a 12-well culture plate following a preincubation period of 20–40 min at 37 °C and 5% CO_2_. The coverslips were washed with Ringer-like solution (RLS) to remove any unbound dye. Subsequently, the coverslips were transferred to a microscope bath chamber containing either RLS or a related antagonist solution. For the fluorescence optic measurements, a fluorescence microscope (Olympus BX50WI; Olympus Europa Holding GmbH, 20097 Hamburg, Germany) equipped with Omikron V. 1.0 software-controlled high-powered fluorescence LED light source (LED-Hub and software by Omikron, 63110 Rodgau–Dudenhoven, Germany) as well as a peristaltic pump P-1 (Pharmacia, London, UK) connected to the bath chamber were used in a dark room. The cellSens Dimension V. 1.16 software (Olympus Europa Holding GmbH, 20097 Hamburg, Germany) was utilized to select the appropriate measurement region, and regions of interest (ROI) (including multiple single cells and a background area) were marked using a polygon ROI graphic software tool (included in the cellSens Dimension V. 1.16 from Olympus, 20097 Hamburg, Germany). The experimental protocol involved stimulating the cells with 340 nm-wavelength (colored green by the software) and 380 nm-wavelength (colored red by the software) light every 5 s with 900 ms (380 nm) and 2.68 s exposure time (340 nm), respectively, over a period of 10 min. Reagents were diluted in RLS. Once a stable baseline was established for approximately 4 min, the RLS was replaced with the corresponding reagent solution. Intracellular free-Ca^2+^ levels were measured at room temperature using fura-2/AM emission at 510 nm. The fluorescence ratio analysis (f340 nm/f380 nm) was calculated using the aforementioned cellSens software from Olympus, 20097 Hamburg, Germany. The fluorescent data files at 340 nm and 380 nm underwent thresholding and filtering, and the resulting data were recorded using a software-controlled digital camera (Olympus XM10 from Olympus, 20097 Hamburg, Germany). It should be noted that all these steps were conducted in a dark room to preserve the stability and fluorescence properties of the fura-2 fluorescent dye. The fluorescence ratios f340/f380 were normalized while maintaining the trends in the trace effects at arbitrary values. For control assays, the values were set to 0.1, achieved using the TIDA V. 5.25 software for Windows (HEKA Electronic, 67466 Lamprecht/Pfalz, Germany). The data were then averaged (with error bars) using SigmaPlot version 12.5 for Windows (Systat Software, Inc., Point Richmond, CA, USA). The results were presented as mean values of the (f340 nm/f380 nm) ratio with standard error mean bars (in both directions) representing the data variability. The number of reads per data point (n-values) was also reported. Finally, GraphPad Prism software version 5.00 for Windows (La Jolla, CA, USA) was employed for data visualization and statistical analysis.

### 4.10. Viability Assay

To obtain a suitable non-toxic concentration of CAP and icilin for the stimulation of the hMGECs, we performed a CellTiter-Glo 3D cell viability assay (Promega, G9681, Walldorf, Germany) according to the manufacturer’s instructions.

### 4.11. Oil Red O Assays

For the oil red O experiments, cells were cultivated in a 48-well plate at a confluence of 0.06 × 10^6^ hMGECs per well. After 24 h, the medium was changed to either 300 µL of proliferation medium or 300 µL of differentiation medium per well. After 24 h of differentiation, hMGECs were incubated (a) without stimulant (=control), (b) medium containing 10 µM CAP in ethanol and (c) medium containing 100 µM icilin in DMSO. The stimulation experiments were carried out in proliferation medium and in differentiation medium for 24 h. The cells were washed three times with PBS at room temperature and conserved in 4% PFA at 4 °C. For the oil red O staining, the cells were washed twice with a.d. and covered with 60% isopropyl for 5 min. The oil red O stock solution (O0625, Sigma-Aldrich, Merck, Germany) was prepared and diluted according to the manufacturer’s instructions and filtered twice before incubating the cells in it for 15 min. After three more washes with aqua dest, filtered hemalaun was added for 1 min before washing the cells for 3 min under flowing water. Aqua dest was added and the cells were photographed using a fluorescence microscope (Keyence BZ-X800E, Osaka, Japan). Ten pictures were taken of each well and lipid contents were analyzed and summarized to one mean value per well using ImageJ software (version 1.54 h). 

### 4.12. Statistical Analysis

Paired data were probed for normality according to the Kolmogorov–Smirnov test, and the Student’s *t*-test assessed statistical significance of paired data if they passed normality. Alternatively, the Wilcoxon matched pairs test was used if they failed normality. Likewise, statistical significance was determined for unpaired data using Student’s *t*-test if they passed normality or using Mann–Whitney U test if they failed normality testing. Probabilities of *p* < 0.05 (indicated by asterisks for paired data (*) and hash tags (#) for unpaired data) were considered significant. Further results were analyzed using ordinary one-way ANOVA tests. Results are expressed as the mean ± SEM and *p*-values below 0.05 were considered significant. Calculations and visualizations were performed using GraphPad Prism software version 5.00 and 9.00 for Windows (La Jolla, CA, USA). 

## Figures and Tables

**Table 1:** Lipid accumulation in hMGECs after TRPV1 and TRPM8 stimulation. Immortalized hMGECs were stimulated for 24 h with CAP (10 µM) or icilin (I, 100 µM) and lipid content was analyzed and compared to unstimulated controls using ORO staining.

n = 12	Lipid Content Compared to a Control	*p*-Value
Prol. hMGECs 10 µM CAP	−2.8 ± 5.74%	>0.05
Prol. hMGECs 100 µM I	−12.1 ± 0.65%	>0.05
Diff. hMGECs 10 µM CAP	+8.8 ± 3.02%	<0.05
Diff. hMGECs 100 µM I	−10.97 ± 1.73%	<0.0001

**Table 2:** Primers used for Reverse Transcriptase Polymerase Chain Reaction and quantitative Real-Time Polymerase Chain Reaction.

RT-PCR	Forward	Reverse	Product Length
Primer			
Human			
*TRPV1*	CTCCTACAACAGCCTGTAC	AAGGCCTTCCTCATGCACT	285 bp
*TRPV2*	CTCTGGTGGCTAGCCTGTCCTGACA	TGGGATCCCGGAGCTTCTCA	228 bp
*TRPV3*	GCTGAAGAAGCGCATCTTTGCA	TCATAGGCCTCCTCTGTGTACT	288 bp
*TRPV4*	TACCTGTGTGCCATGGTCATCT	TGCTATAGGTCCCCGTCAGCTT	379 bp
*TRPM8*	CCTGTTCCTCTTTGCGGTGTGGAT	TCCTCTGAGGTGTCGTTGGCTTT	621 bp
*TRPA1*	GACCACAATGGCTGGACAGCT	GTACCATTGCGTTGAGGGCTGT	541 bp
murine			
*TRPV1*	CCCCCAAAACAGTAGCTTCA	AGCAACACCAGCCCAATTAC	239 bp
*TRPV2*	TGATGAAGGCTGTGCTGAAC	CACCACAGGCTCCTCTTCTC	174 bp
*TRPV3*	AGGCTTCTATTTTGGCGAGACAC	TCCCGAGGACGGTAGTAAGAGAC	623 bp
*TRPV4*	ACAACACCCGAGAGAACACC	TGAACTTGCGAGACAGATGC	221 bp
*TRPM8*	CGACAACTCAGAGGAGATGAGG	GGGATGGGGTAGGACTCTTTG	525 bp
*TRPA1*	GGAGCAGACATCAACAGCAC	GCAGGGGCGACTTCTTATC	393 bp
qPCR			
human			
*TRPV1*	CAGCAGCGAGACCCCTAA	CCTGCAGGAGTCGGTTCA	65 bp
*TRPV4*	CAACAACGACGGCCTCTC	GGATGATGTGCTGAAAGATCC	73 bp
*TRPM8*	GCCAAAGTGAAGAACGACATC	ACACTCATGAACAGCTCAACAG	93 bp

**Table 3:** Antibodies used for Immunofluorescence (IF) and Western blot (WB).

Antibodies	Manufacturer	Product Number	IF	WB
**primary**				
TRPV1	alomonelabs	ACC-030	1:50	1:200
TRPV3	antikoerper-online.de	ABIN863127	1:100	1:1000
TRPV4	alomone labs	ACC-034	1:150	1:200
TRPM8	BIOZOL	LS-B6607	1:50	1:1000
GAPDH	Santa Cruz biotechnology	sc-365062		1:2000
**secondary**				
HRP	Agilent Dako	P0448		1:5000
Alexa Fluor 488	Thermo Fisher Scientific	A11008	1:1000	

## Data Availability

The data presented in this study are available on request from the corresponding author.
